# Serum Apoptosis Markers Related to Liver Damage in Chronic Hepatitis C: sFas as a Marker of Advanced Fibrosis in Children and Adults While M30 of Severe Steatosis Only in Children

**DOI:** 10.1371/journal.pone.0053519

**Published:** 2013-01-11

**Authors:** Pamela Valva, Paola Casciato, Carol Lezama, Marcela Galoppo, Adrián Gadano, Omar Galdame, María Cristina Galoppo, Eduardo Mullen, Elena De Matteo, María Victoria Preciado

**Affiliations:** 1 Laboratory of Molecular Biology, Pathology Division, Hospital de Niños Ricardo Gutiérrez, Buenos Aires, Argentina; 2 Liver Unit, Hospital Italiano de Buenos Aires, Buenos Aires, Argentina; 3 Liver Unit of University of Buenos Aires at Hospital de Niños Ricardo Gutiérrez, Buenos Aires, Argentina; 4 Pathology Division, Hospital Italiano de Buenos Aires, Buenos Aires, Argentina; University of North Carolina School of Medicine, United States of America

## Abstract

**Background:**

Liver biopsy represents the gold standard for evaluating damage and progression in patients with chronic hepatitis C (CHC); however, developing noninvasive tests that can predict liver injury represents a growing medical need. Considering that hepatocyte apoptosis plays a role in CHC pathogenesis; the aim of our study was to evaluate the presence of different apoptosis markers that correlate with liver injury in a cohort of pediatric and adult patients with CHC.

**Methods:**

Liver biopsies and concomitant serum samples from 22 pediatric and 22 adult patients with CHC were analyzed. Histological parameters were evaluated. In serum samples soluble Fas (sFas), caspase activity and caspase-generated neoepitope of the CK-18 proteolytic fragment (M30) were measured.

**Results:**

sFas was associated with fibrosis severity in pediatric (significant fibrosis p = 0.03, advanced fibrosis p = 0.01) and adult patients (advanced fibrosis p = 0.02). M30 levels were elevated in pediatric patients with severe steatosis (p = 0.01) while in adults no relation with any histological variable was observed. Caspase activity levels were higher in pediatric samples with significant fibrosis (p = 0.03) and they were associated with hepatitis severity (p = 0.04) in adult patients. The diagnostic accuracy evaluation demonstrated only a good performance for sFas to evaluate advanced fibrosis both in children (AUROC: 0.812) and adults (AUROC: 0.800) as well as for M30 to determine steatosis severity in children (AUROC: 0.833).

**Conclusions:**

Serum sFas could be considered a possible marker of advanced fibrosis both in pediatric and adult patient with CHC as well as M30 might be a good predictor of steatosis severity in children.

## Introduction

Hepatitis related to Hepatitis C virus (HCV) is a progressive disease that may result in chronic active hepatitis, cirrhosis, and hepatocellular carcinoma. It is estimated that over 200 million people are infected worldwide, while 80% develop a chronic form [1]. It represents a global health problem since there is no vaccine available, the response to current standard of care therapy is limited and liver failure related to chronic hepatitis C (CHC) virus infection is one of the most common reasons for liver transplants [2]. Liver disease seems to be milder in children than in adults; however, the natural history of HCV infection acquired in infancy and childhood remains poorly characterized and the long-term outcome of the disease is still a matter of debate [3].

Although liver biopsy represents the gold standard for evaluating presence, type and stage of liver fibrosis and for characterizing necroinflammation; it remains an expensive and invasive procedure with inherent risks. Thus, it cannot be performed frequently to monitor therapeutic outcomes [4], [5]. Moreover, in children, biopsy is still perceived to carry a higher risk of complications, so it is less accepted than in adults. Therefore, developing noninvasive tests that can accurately predict initial disease stage and progression over time represents a high priority and growing medical need [6], [7].

Several less invasive diagnostic methods are currently being validated as potential tools to determine liver damage, namely serum markers and image methods, but they have not been yet incorporated in clinical practice in most countries [8]. Many authors have proposed multiple indexes based on the combination of biochemical markers with clinical data (i.e. Fib-4, Forms or Fibrotest) or biochemical and clinical markers with fibrosis parameters (i.e. Hepascore, Shasta and Fibrometer) to predict fibrosis stage [9], [10], [11], [12], [13], [14]. Related to that, we have previously studied, in a cohort of pediatric and adult patients, the presence of a pro-fibrogenic cytokine (TGF-ß1) as well as different matrix deposition markers [hyaluronic acid (HA), type III procollagen amino-terminal peptide (PIIINP) and tissue inhibitor of matrix metalloprotein inhibitor-1 (TIMP-1)] related to liver injury during CHC. The results demonstrated that given the diagnostic accuracy of HA, PIIINP, TGF-ß1, their combination may provide a potential useful tool to assess liver fibrosis in adults. On the other hand, in pediatric patients TIMP-1 could be clinically useful for predicting liver fibrosis in patients with CHC [15].

Considering that 1) apoptosis plays a major role in the tissue development and homeostasis and in pathological processes [16]; 2) it has been demonstrated that hepatocyte apoptosis plays a role in liver pathogenesis of CHC; as well as it may be associated with liver fibrogenesis [17], [18], [19], [20]; the aim of our study was to evaluate the presence of different apoptosis markers which correlate with liver injury in a cohort of pediatric and adult patients with CHC infection.

## Methods

### Patients and samples

Twenty two pediatric patients with CHC (8 male, 14 female; range of age at biopsy: 1–17 years, median: 8 years) from Hospital de Niños Ricardo Gutierrez (HNRG) and 22 adult patients (13 male, 9 female; range of age at biopsy: 38–74 years, median: 51 years) from Hospital Italiano de Buenos Aires (HIBA) were enrolled in the present study.

Diagnosis was based on the presence of anti-HCV antibodies in serum at or after 18 months of age and HCV RNA in plasma at one or more separate occasions. Patients had no other causes of liver disease, autoimmune or metabolic disorders, hepatocellular carcinoma and coinfection with hepatitis B virus and/or human immunodeficiency virus. In adult cases, patients with a history of habitual alcohol consumption were excluded (>80 g/day for men and >60 g/day for women). Patients were naïve of treatment. This study has the approval of the Institutional Review Board and the Ethics Board of both HNRG and HIBA and is also in accordance with the Helsinki Declaration of 1975, as revised in 1983. A written informed consent was obtained from all the included adult patients and from parents of pediatric patients after the nature of the procedure had been fully explained.

Formalin-fixed paraffin-embedded liver biopsies and serum samples at time of biopsy were used for histological and serological analysis, respectively. Histological sections were evaluated by two independent pathologists in a blind manner. Inflammatory activity and fibrosis were assessed using the modified Knodell scoring system (Histological Activity Index, HAI) and METAVIR [21]. According to HAI, each biopsy specimen was categorized as minimal (≤3), mild (4–6), moderate (7–12) or severe hepatitis (>12). Presence of lymphoid follicles as well as of bile duct lesion and grade of steatosis were also evaluated. Steatosis was graded as follows: minimal (1–33% of hepatocytes affected), moderate, (>33%–66%) or severe (>66%). Serum AST and ALT levels and genotype were obtained from clinical records. As controls, serum samples from pediatric (n = 9) and adult (n = 9) healthy subjects without known systemic or liver disease and with normal biological liver test as well as absence of anti-HCV antibodies, were included.

In adult cases, liver samples were not obtained from patients diagnosed as having liver cirrhosis based on clinical, biochemical and imaging findings. Although, there are no pediatric specific guidelines about the need for and timing of a liver biopsy in children, the probability of a child undergoing liver biopsy in this study reflected the current practice at our centre, which is based mostly on the national experts consensus [22]. In two pediatric cases, more than one sample was available.

### Quantitative assessment of apoptosis markers

Soluble Fas (sFas), caspase activity and caspase-generated neoepitope of the CK-18 proteolytic fragment (M30) were measured as apoptosis markers.

Serum sFas and M30 were determined by commercial quantitative sandwich enzyme immunoassay technique (*Quantikine Human soluble Fas kit*, R&D Systems Inc; and *M30-Apoptosense ELISA kit*, PEVIVA; respectively) according to the manufacturer's instructions. Serum concentration for each marker was determined from standard curves. Serum sFas was expressed as pg/mL and M30 as U/L.

Serum Caspase activity was determined using a chemiluminescence assay (Casp*ase-Glo Assay*, Promega). Briefly, samples were first diluted 1∶1 in a buffer containing 50 mM Tris-HCl, 10 mM KCl, 5% glycerol, pH 7.4 and incubated with 25 µl of samples or controls diluted with an equal volume of caspase substrate for 3 h at room temperature. Then, the samples' luminescence was measured for 20 seconds in the Luminometer Junior LB 9509 (*Berthold Technologies GmbH & Co. KG*). Results are expressed as RLU. An activity negative control (25 µl buffer 50 mM Tris-HCl, 10 mM KCl, 5% glycerol, pH 7.4) and positive control (25 µl Human Recombinant activated Caspase-3 Protein, Millipore, CHEMICON 0.04 U/µl in the same buffer) were included in each assay.

Each serum marker concentration was assessed in duplicate.

Operators who perform the laboratory tests were blinded for patient's clinical and histological data.

### Statistical analysis

Statistical analysis was performed using GraphPad InStat software, version 3.05. To compare the means between groups, ANOVA or Student's t test were performed. To determine differences between groups not normally distributed, medians were compared using the Mann-Whitney U test or Kruskal Wallis test. Pearson's correlation coefficient was used to measure the degree of association between continuous, normally distributed variables. The degree of association between non-normally distributed variables was assessed using Spearman's nonparametric correlation. To compare categorical variables Fisher's exact Test was applied. P values<0.05 were considered statistically significant. The results are depicted in box plots. Horizontal lines within boxes indicate medians. Horizontal lines outside the boxes represent the 5 and 95 percentiles. Mean is indicated as +.

To assess the ability of the serum apoptosis markers to differentiate hepatitis grade, fibrosis stages and steatosis grade, we calculated the sensitivity and the specificity for each value of each marker and then constructed receiver operating characteristic (ROC) curves by plotting the sensitivity against the reverse specificity at each value. The diagnostic value of each serum marker was assessed by the area under the ROC (AUROC). AUROC of 1.0 is characteristic of an ideal test, whereas 0.5 indicates a test of no diagnostic value. We determined the cut-off value for the diagnosis, as the maximal value at the sum of the sensitivity (Se) and specificity (Sp). The diagnostic accuracy was calculated by sensitivity, specificity and positive and negative predictive values. Area under the ROC, cut off values, positive predictive values (PPV) and negative predictive values (NPV) were determined using the MedCalc demo statistical software (Mariakerke, Belgium).

## Results

### Clinical and liver biopsy findings

Clinical, virological, and histological features of patients are described in Table 1 (pediatric patients) and Table 2 (adult patients).

**Table 1:** Clinical, virological and histological features of pediatric CHC patients.

		Clinical and serological characteristics						Histological characteristics				
Pediatric Patients		Sex	Ages (ys)	Risk factor for HCV infection	Genotype	Transaminases		Knodell	Fibrosis stages #	Lymphoid Follicle	Bile duct damage	Steatosis
						AST (U/L)	ALT (U/L)					
1		M	13	T	1a	47	43	8(5+3)	F2	no	no	absent
2		F	14	T	1b	55	86	11(9+2)	F1	no	no	minimal
3		F	4	V	1a/c	46	34	10(7+3)	F2	yes	yes	absent
4		F	17	T	1a/c	39	43	8(4+4)	F3	no	yes	moderate
5		M	4	V	1a/c	84	97	10(9+1)	F1	yes	yes	severe
6	BxI	F	6	V	1a/c	13	11	7(3+4)	F3	no	no	absent
	BxII		13			23	21	8(5+3)	F2	no	yes	minimal
7		F	16	Unknown	1a/c	30	41	12(10+2)	F1	yes	yes	minimal
8	BxI	M	3	V	1a/c	71	91	6(5+1)	F1	yes	yes	minimal
	BxII		6			314	364	11(8+3)	F2	no	yes	severe
	BxIII		13			225	260	21(16+5)	F3	no	yes	moderate
9		F	6	V	1a/c	35	50	8(7+1)	F1	yes	yes	minimal
10		F	8	Unknown	1a	41	38	9(7+2)	F1	yes	yes	absent
11		M	13	Unknown	ND	56	71	10(7+3)	F2	no	yes	absent
12		F	17	V	1a/c	21	11	6(3+3)	F2	no	yes	minimal
13		F	3	V	1a/c	84	137	6(5+1)	F1	no	yes	moderate
14		F	3	V	1b	65	75	9(6+3)	F2	yes	yes	moderate
15		F	1	V	4	57	33	11(7+4)	F3	yes	yes	absent
16		F	17	T	1a/c	22	16	10(7+3)	F2	no	yes	minimal
17		M	1	T	1b	159	213	14(11+3)	F2	yes	yes	minimal
18		F	8	T	1b	10	12	6(5+1)	F1	no	no	absent
19		M	15	T	ND	58	76	15(10+5)	F3	no	yes	absent
20		F	6	V	1a/c	56	55	6(5+1)	F1	no	yes	severe
21		M	15	T	1b	20	24	13(10+3)	F2	yes	yes	absent
22		M	12	Unknown	1a/c	83	113	11 (8+3)	F2	no	yes	minimal

F: female, M: male. ND: not determined Bx I, Bx II, Bx III denote: multiple liver biopsies. Risk factor for HCV infection: T: transfusion, V: vertical transmission.

ALT: alanine aminotransferase; AST: aspartate aminotransferase. Normal ALT and AST levels were ≤32 and ≤48 IU/L, respectively when test was done at 37°C.

# Fibrosis stages according METAVIR.

**Table 2:** Clinical, virological and histological features of adult CHC patients.

	Clinical and serological characteristics						Histological characteristics*				
Adult Patients	Sex	Ages (ys)	Risk factor for HCV infection	Genotype	Transaminases		Knodell	Fibrosis stages #	Lymphoid Follicle	Bile duct damage	Steatosis
					AST (U/L)	ALT (U/L)					
1	M	38	Unknown	1b	82	89	6(5+1)	F1	yes	yes	absent
2	F	52	T	1b	45	52	8(6+2)	F1	yes	yes	minimal
3	M	42	Unknown	1a	44	56	9(8+1)	F1	yes	yes	absent
4	F	62	T	1a	42	32	11(7+4)	F3	no	yes	moderate
5	M	48	DA	1b	40	63	10(7+3)	F2	no	yes	severe
6	M	40	DA	1a	34	45	10(6+4)	F3	yes	yes	moderate
7	M	47	Unknown	1a	29	54	8(6+2)	F1	yes	yes	minimal
8	M	40	DA	2a	63	79	4(4+0)	F0	yes	no	absent
9	M	41	DA	3a	79	86	7(4+3)	F2	no	yes	absent
10	F	61	Unknown	1a	31	28	17(12+5)	F3	yes	yes	absent
11	F	72	Unknown	1a/c	52	71	9(6+3)	F2	yes	yes	minimal
12	F	74	T	1*	106	85	15(12+3)	F2	yes	yes	absent
13	M	62	Unknown	1b	22	32	10(7+3)	F2	no	yes	absent
14	F	55	Unknown	3b	49	60	8(6+2)	F1	yes	yes	severe
15	F	67	Unknown	2a	28	26	6(6+0)	F0	yes	yes	minimal
16	F	41	Unknown	1a	192	105	6(2+4)	F3	yes	yes	absent
17	M	51	OE	1b	65	74	5(4+0)	F0	yes	yes	absent
18	M	51	DA	1a	73	109	10(7+3)	F2	yes	yes	minimal
19	F	67	T	1b	106	103	13(9+4)	F3	yes	yes	absent
20	M	73	Unknown	1b	31	42	8(6+2)	F1	yes	yes	absent
21	M	41	DA	1a	32	50	10(7+3)	F2	yes	yes	severe
22	M	47	DA	3	38	59	3(2+1)	F1	yes	yes	minimal

F: female, M: male.

* Subtype not determined Risk factor for HCV infection: T: transfusion, DA: drug abuse, OE: occupational exposure.

ALT: alanine aminotransferase; AST: aspartate aminotransferase. Normal ALT and AST levels were ≤40 and ≤42 IU/L, respectively when test was done at 37°C.

# Fibrosis stages according METAVIR.

In both groups HCV genotype 1 was predominant, 86% in pediatric cases and 77% in adults. The risk factors for HCV infection in children were 46% vertical transmission, 36% transfusion and 18% unknown. In adults, seven cases (32%) had a history of injecting drug abuse, one case (5%) described an occupational exposure to infected blood, four (18%) a transfusion as a risk factor and 10 (45%) an unknown source for transmission. The aspartate aminotransferase (AST) and alanine aminotransferase (ALT) levels at time of biopsy, considering multiple biopsies from the same patient in 2 pediatric cases, were elevated in 52% and 76% serum samples of pediatric patients, respectively and in 59% and 77% serum samples of adult patients as well.

Eighteen percent of pediatric biopsies showed moderate or severe hepatitis, while concerning fibrosis, bridging fibrosis (stage 2 of METAVIR) was predominant among studied biopsies (44%). In adult cases, moderate or severe hepatitis were present in 73% of biopsies and the fibrosis profile displayed was 32% stage 1, 32% stage 2 and 23% stage 3. Finally, 3 adult patients showed absence of fibrosis. The prevalence of significant fibrosis (F≥2) and advanced fibrosis (F≥3) in the pediatric cohort were 64% and 20%, respectively; meanwhile it was 54% F≥2 and 23% F≥3 in adults. Lymphoid follicles, characteristic of CHC in adults, were present in 40% of pediatric and 82% of adult specimens, whereas bile duct lesions were observed in 83% of pediatric and 95% of adult samples. Hepatocellular fat accumulation, typically a mixture of small and large droplet fat, was present in both series (64% of pediatric and 50% of adult cases). Minimal steatosis was observed in 36%, moderate in 12% and severe in 16% of pediatric biopsies; meanwhile in adults minimal, moderate and severe steatosis were present in 27%, 9% and 14%, respectively. The comparative statistical analysis of all histological parameters between pediatric and adult studied patients did not showed any significant difference except for lymphoid follicles (p = 0.01). However, it should be taken into account that adult cases with liver cirrhosis based on clinical, biochemical and imaging findings were not biopsied.

Finally, aminotransferase values were not associated to any parameter of histological liver damage.

### Quantitative assessment of sFas, caspase activity and M30

Apoptosis markers were first compared between patients with CHC and healthy subjects. Then in a further analysis CHC patients apoptosis markers were related to histological parameters of liver injury, particularly fibrosis, hepatitis and steatosis severity.

**Figure 1:**
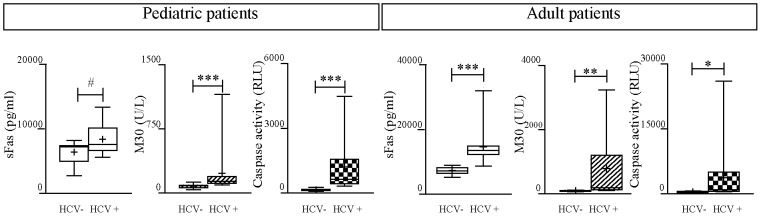
Apoptosis markers in serum samples from CHC patients vs healthy subjects. *p<0.05; ** p<0.001; ***p<0.0001;·^#^ trend of association p<0.07.

As it is shown in Figure 1, apoptosis markers were significantly increased in serum samples from both pediatric and adult patients with CHC compared to healthy subjects, except for sFas levels in CHC pediatric patient samples which only showed a trend of association (p = 0.07).

**Figure 2:**
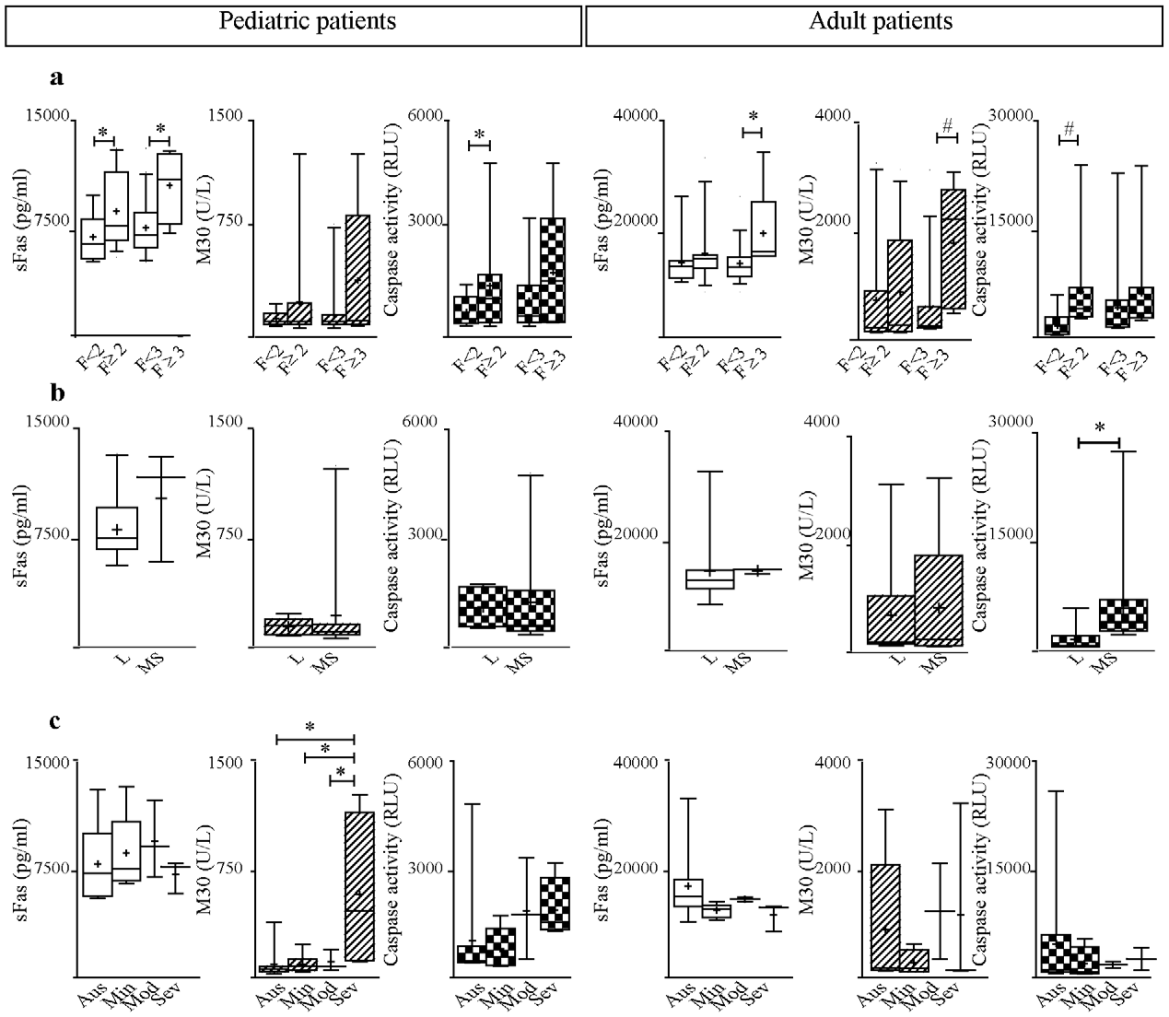
Serum markers related to liver damage in CHC patients. a) fibrosis, b) hepatitis and c) steatosis severity. Fibrosis stages according METAVIR. Significant fibrosis (F≥2) and advanced fibrosis (F≥3). * p<0.05;·^#^ trend of association p<0.08.

With regard to liver damage, sFas was associated with fibrosis severity in both pediatric and adult CHC patients. It was significantly increased in children with significant fibrosis (p = 0.03) and advanced fibrosis (p = 0.01), and in adults with advanced fibrosis (p = 0.02) (Figure 2a). It is worth mentioning that serum sFas levels of pediatric patients with mild fibrosis stages (F1 and F2) showed no significant differences compared with those levels of pediatric healthy subjects. It is in accordance with most noninvasive makers that offer most reliable results at the extreme fibrosis stages. Finally, sFas was not associated with hepatitis severity or steatosis degree in any of the studied age groups (Figure 2b, c).

The M30 association profile related to the histological parameters was different between children and adults. In pediatric cases, M30 levels were elevated in patients with severe steatosis (p = 0.01) (Figure 2c) while in adults no relation with this histological variable was observed. Concerning fibrosis a trend of association between this marker and advanced fibrosis (p = 0.05) in adults was depicted (Figure 2a). Finally, there were no significant differences in serum M30 levels with respect to hepatitis in none of the studied age groups (Figure 2b).

The caspase activity profile in relation to fibrosis severity was similar to that observed for sFas and M30 in both populations. Caspase activity levels were higher in those cases with significant and advanced fibrosis; however, the difference turned out to be statistically significant only in samples from children with significant fibrosis (p = 0.03). In the adult cohort only a trend of association with significant fibrosis was observed (p = 0.08). In turn, in serum samples from adult patients caspase activity was associated with hepatitis severity (p = 0.04) (Figure 2b). No association between steatosis degree and caspase activity was observed in any of the studied groups (Figure 2c).

### Diagnostic performance of apoptosis markers

The evaluation of the diagnostic performance was only assessed for those apoptosis markers which had shown to be associated with histological injury variables. Tables 3, 4 and 5 show the diagnostic accuracy of each marker by means of the sensitivity, specificity, positive and negative predictive values.

**Table 3:** Diagnostic accuracy of sFas for significant and advance fibrosis in CHC patients.

SIGNIFICANT FIBROSIS (F≥2)							
	AUROC	95% CI	Cut off *	Se%	Sp%	PPV	NPV
**PEDIATRIC PATIENTS**	0.719	0.500–0.881	6815.14	86.67	55.56	76.5	71.4

ADVANCED FIBROSIS (F≥3)							
	AUROC	95% CI	Cut off *	Se%	Sp%	PPV	NPV
**PEDIATRIC PATIENTS**	0.812	0.602–0.941	7416.56	100	55	30.8	100
**ADULT PATIENTS**	0.800	0.577–0.938	13806.67	100	70.60	50	100

* pg/ml.

**Table 4:** Diagnostic accuracy of M30 for steatosis severity in pediatric CHC patients.

SEVERE STEATOSIS							
	AUROC	95% CI	Cut off *	Se%	Sp%	PPV	NPV
**PEDIATRIC PATIENTS**	0.833	0.634–0.951	114.53	100	57.14	30.8	100

* U/L.

**Table 5:** Diagnostic accuracy of Caspase activity for significant fibrosis and moderate/severe hepatitis in CHC patients.

SIGNIFICANT FIBROSIS (F≥2)							
	AUROC	95% CI	Cut off *	Se%	Sp%	PPV	NPV
**PEDIATRIC PATIENTS**	0.656	0.436–0.836	1490	37.5	100	100	44.4

MODERATE/SEVERE HEPATITIS							
	AUROC	95% CI	Cut off *	Se%	Sp%	PPV	NPV
**ADULT PATIENTS**	0.744	0.510–0.907	676	86.67	83.33	92.9	71.4

* RLU.

When considering a less invasive test as good as a liver biopsy to evaluate liver damage, the AUROC of the marker must be equal to or greater than 0.800 [6]. Therefore, in this study, only those markers which AUROC was greater than this value were taken into account. Thus, sFas quantification demonstrated a limited utility as a less invasive marker of significant fibrosis in pediatric patients (AUROC: 0.719), but it could be considered a possible marker of advanced fibrosis both in children and adults (children AUROC: 0.812, NPV 100%, adults AUROC: 0.800, NPV 100%) (Table 3). On the other hand, M30 showed an AUROC of 0.833 and a high NPV (100%) indicating that it might be a good marker of steatosis severity in pediatric patients (Table 4). Finally, despite the observed association between caspase activity and significant fibrosis stage in pediatric patients as well as moderate/severe hepatitis in adults, this marker would not be useful as a less invasive indicator of liver damage. Although, both specificity and PPV were high, AUROC values were very low (Table 5).

The cut off value for sFas to differentiate advanced fibrosis in pediatric patients was 7416.56 pg/ml (100% Se, 55% Sp), whereas in adults it was 13806.67 pg/ml (100% Se, 70.60% Sp) (Table 3). Serum M30 cut off value for diagnosis of severe steatosis in pediatric patients was 114.53 U/L (100% Se, 57.14% Sp) (Table 4).

## Discussion

Apoptosis has been implicated in the pathogenesis of a number of hepatic disorders, including viral hepatitis, autoimmune diseases, non-alcoholic steatohepatitis, alcohol-induced injury, cholestasis and hepatocellular cancer [23], [24], [25], [26], [27]. There is increasing evidence suggesting that liver cell damage in CHC is mediated by apoptosis induction, which has been proposed in view of pathomorphologic features of infected hepatocytes [17], [24], [28], [29]. Several viral proteins display either apoptotic or antiapoptotic features according to the model under study [18], [30]; in turn, both *in vitro* studies or *in vivo* models with whole virus demonstrated its ability to induce apoptosis. Our previous study demonstrated that apoptosis of hepatocytes is a prominent feature observed in liver biopsies of patients with CHC and that it is related to the pathogenesis of the disease [20]. Here, we evaluated whether the apoptosis markers were a remarkable feature in serum samples of pediatric and adult CHC patients and analyzed their relation to liver damage. According to available data and their importance in the pathogenesis, three components of the apoptosis process were selected for evaluation: **1)** *sFas*, since it has been proposed that apoptosis triggered by Fas/FasL is a major cause of hepatocyte damage together with the observed high Fas/FasL expression levels which correlate with liver injury during CHC [31], [32], [33], [34], [35]; **2)** *caspase activity*, since it has been reported that caspases are activated in HCV infected patient hepatocytes and are responsible for most of the morphological changes of the apoptotic cells [36]; and **3)** *M30*, since CK18 is the major intermediate filament of hepatocytes which is, in turn, a caspase substrate whose cleavage contribute to cellular collapse during apoptosis.

According to previously published data, this study showed that serum sFas levels were high in patients with CHC [31], [37], [38], [39], [40], [41], [42]; however, the clinical relevance of circulating sFas is not completely understood. As described above, Fas/FasL interaction is the primary initiator of the extrinsic apoptosis pathway in the liver, hence the elimination of apoptotic bodies in pathological conditions may induce an inflammatory reaction with the consequent activation of stellate cells, which in turn favors the development of liver fibrosis [43]. Several authors postulated that sFas is associated with liver damage severity because significantly increased sFas levels were observed in patients with terminal disease stages such as cirrhosis and HCC [31], [44]. In turn, Toyoda et al reported that sFas levels in CHC patients correlated with hepatitis severity [45]. Kakiuchi et al corroborated this result, but on the other hand, reported that this marker is not associated with fibrosis severity [40]. In contrast, the results herein indicated that sFas was not related to hepatitis severity, but instead was associated with fibrosis severity. It is important to note that despite the higher sFas levels detected in CHC children compared to healthy subjects, only a trend of association was observed. A possible explanation for this observation would be that sFas levels in pediatric patients with mild fibrosis stages were similar to those of pediatric healthy subjects; furthermore in our studied series the group of children with severe fibrosis was small since a severe stage of fibrosis is not commonly present in CHC patients during infancy and childhood.

With respect to activation of caspases, the current results corroborate that apoptosis is an important event in CHC, as we have previously observed when investigating caspase activity and M30 expression in liver samples from CHC patients [20]. The assessment of caspases activation and M30 showed high values in the two types of samples tested, but in none of the two age groups these markers detected on liver biopsies correlated with their corresponding marker in serum. Since liver and serum are distinct compartments, one possible explanation for the lack of correlation may be that apoptotic cells are rapidly eliminated and therefore, would not be detected in biopsy. Meanwhile, although the exact mechanism of secretion of M30 in the blood has not been determined yet, it is postulated that serum M30 is released as a result of necrosis secondary to apoptosis. In favor of the latter, we observed correlations between M30 and terminal apoptotic cells (TUNEL+ cells) in both pediatric and adult patients (pediatric patients r = 0.533 p = 0.02; adult patients r = 0.535 p = 0.02) (for technical information see Methods S1).

According to that previously described for adult cohorts [28], M30 levels and caspase activity were significantly increased in serum samples from pediatric and adult patients with respect to control subjects. However, their relation to liver damage is still controversial. Bantel et al found that serum M30 quantification is a highly sensitive method to early detect fibrosis severity [28]. They observed that M30 levels were associated with more severe stages of fibrosis only in patients with normal transaminase values, but no association between M30 and either hepatitis or fibrosis severity in general adult CHC patients was found. On the other hand, Seidel et al found that both M30 and caspase activity were elevated in adult patients with severe steatosis [46]. Finally, Papatheodoridis et al found that M30 is associated with global liver damage severity, because its levels correlated with hepatitis severity, fibrosis and steatosis [47]. In contrast, Joka et al describe that M65, another epitope which is present in both caspase-cleaved and uncleaved CK-18, is more sensitive and specific than M30 for the detection of lower fibrosis stages and steatosis severity in many forms of chronic liver disease, including CHC; although M65 and M30 were not individually analyzed in the context of each disease etiology [48]. In our report each marker showed a different association profile related to liver damage in each of the studied cohorts. Higher levels of caspase activity were observed in adult cases with more severe hepatitis as well as in children with significant fibrosis. While M30 showed a trend of association with advanced fibrosis, it did not correlate with steatosis degree in adult patients. Concerning children, this marker was significantly increased in cases with severe steatosis. This last finding is particularly important since M30 is being widely studied as a marker of the steatosis severity both in pediatric and adult patients with Non Alcoholic Steatohepatitis [49], [50], [51], [52], [53], [54].

A major clinical challenge is finding the best means for evaluating liver impairment in the increasing number of CHC infected patients [2], [9], [55]. Prognosis and treatment of CHC are partly dependent on the assessment of histological activity, namely cell necrosis and inflammation, and on the degree of liver fibrosis. These parameters have so far been provided by liver biopsy, because conventional laboratory tests are unable to precisely evaluate liver lesions. Biopsy, due to its limitations and risks, is no longer considered mandatory as the 1st-line indicator of liver injury in CHC patients [56], [57], [58], [59]. In addition to the risks related to an invasive procedure, liver biopsy has been associated with sampling errors mostly due to suboptimal biopsy size [60], [61], [62]. To avoid these pitfalls, several markers have been proposed as noninvasive alternatives for predicting liver damage; but few, particularly those which combine clinical and biochemical parameters, have been applied to pediatric patients [63], [64]. In this study, the observed relationships between sFas, M30 and caspase activity and liver damage prompted us to assess the diagnostic value of apoptosis markers as potential indicators of liver damage.

Herein, based on AUROC values it was demonstrated that sFas could be a marker of advanced fibrosis both in children and adults and, in turn, M30 could be a good predictor of steatosis severity in children. However, despite the observed association between caspase activity and significant fibrosis in children as well as with hepatitis severity in adult patients, this marker would not be useful as a less invasive indicator of liver damage. Unfortunately, although there are many studies that evaluate these serum apoptosis markers in CHC patients related to liver damage, they do not assess their diagnostic value. This makes it impossible to compare the determined diagnostic accuracy of these markers for the diagnosis of liver injury severity with other reports.

There are several articles that analyze AST-to-platelet ratio (APRI) and AST-to-ALT ratio (AAR) as surrogate indirect serum markers of liver fibrosis. As we previously describe when assessed in our cohorts [15], these approaches did not improve the diagnostic accuracy performance of sFas in pediatric series, since neither APRI nor AAR reached the 0.800 AUROC value proposed to be enough for staging fibrosis. In adults APRI showed a low performance which does not reach the 0.800 AUROC value, while AAR diagnostic value is comparable with the sFas one to predict advanced fibrosis (Table S1).

It should not be ignored that the present study has certain limitations. First, this was in fact a retrospective study, with a quite limited number of cases, so this makes it difficult to validate the utility of serum markers. Second, due to medical management protocols from our institutions, pediatric patients without liver fibrosis (F0) and adults with cirrhosis or hepatic decompensation were not available for this study. Third, since we did not take into account biopsy length and fragmentation, the potential for sampling error and understaging of fibrosis remains possible. Anyway, the molecules here proposed turned out to be easily measurable markers, which can be interpreted in a simple manner. The study of a larger number of cases, perhaps in a multicenter study, will confirm the results obtained in this work and discuss the possibility of adding apoptosis markers to panels that included matrix deposition, clinical and biochemical parameters. Taking into account our previous results on fibrogenesis process direct markers (15) (Table S1), we propose the addition of apoptosis markers, particularly sFas combined with TIMP-1 in pediatric patients and sFas with TGF-ß1, HA, PIIINP in adult patients to more accurately assess liver fibrosis severity.

In conclusion, serum sFas could be considered a possible marker of advanced fibrosis both in pediatric and adult patient with CHC as well as M30 could be a good predictor of steatosis severity in children. Perhaps if these parameters are validated in the near future, they could be easily performed and interpreted and, therefore, could be potentially translatable to the bedside.

## Supporting Information

Methods S1 Technical information about immunohistochemial assays.
(DOC)

Table S1 AUROC for advance fibrosis.
(DOC)
