# Supplementary material for: Serum Apoptosis Markers Related to Liver Damage in Chronic Hepatitis C: sFas as a Marker of Advanced Fibrosis in Children and Adults While M30 of Severe Steatosis Only in Children
Source: PLoS One. 2013 Jan 11;8(1):e53519. doi: 10.1371/journal.pone.0053519 (PMC3543432; doi:10.1371/journal.pone.0053519)
Supplement: Methods S1 — Technical information about immunohistochemial assays. (DOC) [file pone.0053519.s001.doc]

**Samples**

Formalin-fixed and paraffin-embedded liver samples were used for histological analysis as well as for immunodetection of different apoptosis markers.

**Immunohistochemical Analysis (IHC)**

Early and late apoptosis markers were evaluated; namely, 1) activation of caspase-3, a major effector caspase, 2) M30, since CK18 is the major intermediate filament of hepatocytes which is, in turn, a caspase substrate whose cleavage contribute to cellular collapse during apoptosis, and 3) DNA fragmentation, the final and irreversible event in the process of apoptosis.

Tissue sections were deparaffinized in xylene and rehydrated through decreasing concentrations of ethanol. For optimal epitope retrieval, sections were treated with sodium citrate buffer (0.01 M, pH 6). Endogenous biotin was blocked with Biotin Blocking System (DakoCytomation, California, USA). Sections were then incubated with primary antibodies to: caspase-3-mediated cleavage generated neoepitope of CK18 (M30) (Roche, Mannheim, Germany) and activated caspase-3 (R&D systems, Minneapolis, USA). Immunohistochemical staining was obtained by applying the streptavidin-biotin complex–peroxidase system and substrate-chromogen reagent (LSAB+System-HRP and DAB; DakoCytomation, California, USA). IHC was selective for each marker, in that reactive products were not observed in the absence of the respective primary antibody. As positive controls, previously studied pediatric non alcoholic steatohepatitis and chronic hepatitis B virus infection liver biopsy samples positive for both activated caspase-3 and M30 were used. Isotype control was performed.

**TUNEL Staining**

DNA fragmentation was visualized using *ApopTag® Plus Fluorescein In Situ Apoptosis Detection Kit* (CHEMICON International, Inc; Billerica, MA, USA) according to the manufacturer’s instructions. For counterstaining and mounting DAPI in PBS-glycerol (DAPI/*antifade Solution*, CHEMICON International, Inc; Billerica, MA, USA) was used.

**Quantification**

Immunostained and total hepatocytes were counted in 20 high-power fields (1000×).
