# Supplementary material for: Serum Apoptosis Markers Related to Liver Damage in Chronic Hepatitis C: sFas as a Marker of Advanced Fibrosis in Children and Adults While M30 of Severe Steatosis Only in Children
Source: PLoS One. 2013 Jan 11;8(1):e53519. doi: 10.1371/journal.pone.0053519 (PMC3543432; doi:10.1371/journal.pone.0053519)
Supplement: Table S1 — AUROC for advance fibrosis. (DOC) [file pone.0053519.s002.doc]

Table S1: AUROC for advance fibrosis.

AAR: aspartate aminotranferase-to-alanine aminotranferase

|  | **ADVANCED FIBROSIS (F≥3)** | |
| --- | --- | --- |
|  | **AUROC** | **95% CI** |
| **PEDIATRIC PATIENTS** |  |  |
| TIMP-1 | 0.800 | 0.593-0.932 |
| AAR | 0.650 | 0.435-0.828 |
| APRI | 0.600 | 0.366-0.804 |
| **ADULT PATIENTS** |  |  |
| HA | 0.929 | 0.736-0.994 |
| PIIINP | 0.894 | 0.689-0.984 |
| TGF-ß1 | 0.835 | 0.617-0.957 |
| AAR | 0.859 | 0.645-0.969 |
| APRI | 0.692 | 0.427-0.888 |

APRI: aspartate aminotranferase-to-platelet ratio
